# Contrast of Real-Time Fluorescent PCR Methods for Detection of *Escherichia coli* O157:H7 and of Introducing an Internal Amplification Control

**DOI:** 10.3390/microorganisms7080230

**Published:** 2019-07-31

**Authors:** Xihong Zhao, Jing Xia, Yao Liu

**Affiliations:** 1 Research Center for Environmental Ecology and Engineering, Key Laboratory for Green Chemical Process of Ministry of Education, Key Laboratory for Hubei Novel Reactor and Green Chemical Technology, School of Environmental Ecology and Biological Engineering, Wuhan Institute of Technology, Wuhan 430205, China; 2 School of Pharmacy and Food Science, Zhuhai College of Jilin University, Zhuhai 519041, China

**Keywords:** real-time fluorescent PCR, *Escherichia coli* O157:H7, internal amplification control, rapid detection, foodborne pathogens

## Abstract

Various constituents in food specimens can inhibit the PCR assay and lead to false-negative results. An internal amplification control was employed to monitor the presence of false-negative results in PCR amplification. In this study, the objectives were to compare the real-time PCR-based method by introducing a competitive internal amplification control (IAC) for the detection of *Escherichia* O157:H7 with respect to the specificity of the primers and probes, analytical sensitivity, and detection limits of contamination-simulated drinking water. Additionally, we optimized the real-time fluorescent PCR detection system for *E. coli* O157:H7. The specificity of primers and probes designed for the *rfbE* gene was evaluated using four kinds of bacterial strains, including *E. coli* O157:H7, *Staphylococcus aureus*, *Salmonella* and *Listeria monocytogenes* strains. The real time PCR assay unambiguously distinguished the *E. coli* O157:H7 strains after 16 cycles. Simultaneously, the lowest detection limit for *E. coli* O157:H7 in water samples introducing the IAC was 10^4^ CFU/mL. The analytical sensitivity in water samples had no influence on the detection limit compared with that of pure cultures. The inclusion of an internal amplification control in the real-time PCR assay presented a positive IAC amplification signal in artificially simulated water samples. These results indicated that real-time fluorescent PCR combined with the IAC possessed good characteristics of stability, sensitivity, and specificity. Consequently, the adjusted methods have the potential to support the fast and sensitive detection of *E. coli* O157:H7, enabling accurate quantification and preventing false negative results in *E. coli* O157:H7 contaminated samples.

## 1. Introduction

Enterohemorrhagic *Escherichia coli* (EHEC) O157:H7 is a clinically important foodborne and waterborne pathogen [1]. Infection with EHEC O157:H7 can lead to a wide range of clinical manifestations, including asymptomatic infections, mild diarrhea, or severe diseases such as hemorrhagic colitis and hemolytic uremic syndrome [2]. The main virulence factor of EHEC O157:H7, such as the production of Shiga toxins (*Stx*), is located on the pathogenicity island and intestinal cell exfoliation site through signal transduction and close adhesion [3]. The pathogenic mechanism of EHEC O157:H7 is mediated by a series of effectors which are secreted by encoding the translocated intimin receptor (Tir), bacterial encoded receptor, as well as Type III secretory system [4]. Historically, serotype O157 has been the most investigated, even though other serotypes have been involved in several epidemic situations. Between 1978 and 2006, a large number of cases of EHEC O157:H7 infection were reported in Japan, Germany, the United States, and China [5,6,7]. Until now, standardized procedures to detect the presence of these pathogens in food samples have depended on microbiological culturing methods, taking five days to get results [8]. Meanwhile, the traditional culture-based methods usually include an agglutination assay (detecting the O157 or H7 antigen) that is not specific, since the O157 and H7 antigens exist in other *E. coli* species. These antibodies can also cross-react with other *E. coli* serotypes and other members of the Enterobacteriaceae family [9]. In order to reduce the analysis time and improve the rapid, sensitive, and specific detection of pathogens, alternative techniques like molecular methods have been applied for detection from various sample types [10].

General PCR is based on the amplification of species-specific genes or genes related to pathogenicity or virulence. The pathogenic *E. coli* O157:H7 contamination in samples can be rapidly detected together with the results of gel electrophoresis [11]. Recently, the detection of viable but non-culturable (VBNC) *E. coli* O157:H7 in food products and raw materials [12,13] was accomplished by propidium monoazide (PMA)-PCR techniques when samples were preprocessed with PMA, which passed through the cell membrane of dead bacteria and forms covalent carbon-nitrogen bonds with intracellular DNA, and allowed for the determination of dead bacterial DNA [14]. Moreover, real-time PCR is widely applied for bacterial detection and quantification of the initial target DNA of foodborne pathogens [15,16]. Unfortunately, amplification efficiencies can be inhibited due to the presence of inhibitory factors, including food matrix residues, organic solvents from DNA extraction, or other unknown factors. Even when using external controls, quantification doesn’t always provide the correct calculation of the initial target in each sample. The risk of false-negative results is still one limitation that cannot be ignored [17]. To address this problem, the relevant literature reported that the minimum detection limit was reduced to 5 CFU/g by removing possible PCR inhibitors using activated carbon coated with bentonite [18]. However, it is not known whether the introduction of new substances has an effect on the accuracy of the detection limit. To eliminate this drawback, another nucleic acid sequence was suggested to serve as an internal amplification control (IAC) to monitor amplification processes during PCR and to distinguish false-negatives from true-negative results [19,20].

In this study, firstly, a real-time fluorescent PCR-IAC-based method was performed for the specific detection of *E. coli* O157:H7. The applicability of the method was determined with respect to sensitivity and accuracy. Secondly, a competitive internal amplification control (IAC) system, which shared the same primer (a compound primer containing target genes) was constructed to eliminate false negative results. Its influence on the sensitivity, precision and accuracy was respectively evaluated. Finally, the applicability of the quantification of initial target DNA using the real-time fluorescent PCR-IAC-based method was investigated in artificially contamination-simulated water samples, and both the quantification threshold and detection limit of *E. coli* O157:H7 were evaluated.

## 2. Materials and Methods

### 2.1. Design of Primers and TaqMan Probes

The target gene used in this experiment is based on the highly conserved *rfbE* gene in *E. coli* O157:H7. The primer sequence was designed by Primer premier 5.0 software (http://www.premierbiosoft.com/primerdesign/). PCR amplification products were submitted to electrophoresis on 2% agarose gel (Gene Tech, Co., Ltd., Shanghai, China) with 158 bp ladder and visualized by Golden view staining (Sigma, America). In addition, with regard to the selection of TaqMan probes, the molecular beacons employed a double labeled probe which included 5’ end tagged reporter group with 6-carboxy-fluorescein (FAM) and 3’ end labeled quenching group BHQ_1_. The TaqMan probe D-P (5′ TGCAGATAAACTCATCGAAACAAGGCC 3′) of the *rfbE* gene was designed to specifically hybridize to the *rfbE*-coding DNA at 56.8 °C up to 64.8 °C, and was labeled at the 5′ end with FAM and at the 3′ end with BHQ_1_. The design principles of TaqMan probe primers are as follows: primarily, the melting temperature of the TaqMan probe is 5–10 degrees higher than that of the primer. Generally speaking, the length of the probe is less than 30 nucleotides. The 5’ end of the probe cannot be base G because the fluorescence signal can be quenched by base G. Further, the optimum GC content ranged from 30% to 80%, and the content of G is higher than that of C. Ultimately, the location of the probe design is as close as possible to the upstream primers.

The sequences of primers and probes for the *hly* gene detection of *Listeria monocytogenes* were derived from the literature [21]. Both ends of a 64 bp conserved sequence (*hly* gene) were connected with the primer sequence of the *rfbE* gene for *E. coli* O157:H7 and used to develop an IAC for real-time PCR. The TaqMan probe IAC-P (Cy5-CGCCTGCAAGTCCTAAGACGCCA- BHQ2) was labeled at the 5′ end with Cy5 and at the 3′ end with BHQ_2_. Finally, all primers and probes (Table 1) have been constructed using specific matching and blast analysis at the National Centre for Biotechnology Information (NCBI) (http://www.ncbi.nlm.nih.gov/blast/) and were synthesized (Stargene, Co., Ltd., Wuhan, China).

### 2.2. Bacterial Strains and Growth Conditions

The *E. coli* O157:H7 (ATCC 43895), *S. aureus* (ATCC 13565), *Salmonella* (ATCC 13076) and *Listeria monocytogenes* (ATCC 19115) strains were kindly provided by the Food Science and Engineering Microbiology Laboratory of Wuhan Institute of Technology. The four kinds of strains were placed in 20% glycerol (Guoyao, Co., Ltd., Shanghai, China) and stored at −80 °C, were inoculated on LB agar medium (OXOID, Britain) by the parallel scribing method [22], and cultured in a 37 °C incubator for 24 h. Single colonies that grew well on the LB agar medium were picked into LB liquid medium and grown overnight at 37 °C with shaking at 200 rpm, and then a 10-fold dilution series was made ranging from 10^8^ CFU/mL down to 10^1^ CFU/mL by using LB liquid medium. The bacterial suspension was placed in a refrigerator (Gree, Co., Ltd., Guangdong, China) at 4 °C until used.

### 2.3. DNA Extraction of E. coli O157:H7

To analyze and compare the influence on the DNA detection sensitivity between routine PCR (BIORAD, Shanghai, China) and fluorescent quantitative PCR (BIORAD, Shanghai, China), two methods—boiling water and a rapid extraction kit for bacterial genomic DNA (Sangon Biotech, Co., Ltd., Shanghai, China) [23,24]—were used to extract DNA from *E. coli* O157:H7 suspensions.

The DNA extraction by boiling-water procedure used is the following: 1-mL *E. coli* O157:H7 suspension was centrifuged at 8000× *g* for 8 min at room temperature. Subsequently, the pellet was resuspended in 2 mM EDTA solution (500 μL) (Shengxiao, Co., Ltd., Zhejiang, China), and thoroughly mixed (2800 rpm for 1 min) until the bottom cells were completely dispersed in the liquid. Thenceforth, the solution was placed in boiling water at 100 °C for 10 min, and then immediately put on ice for 10 min. Finally, the liquid was centrifuged at 10,000× *g* for 10 min at 4℃ to collect the supernatant. The supernatant was placed in a refrigerator at 4 °C for later use.

As for DNA extraction using the commercial kit (Sangon Biotech, Co., Ltd., Shanghai, China), the procedures were performed according to the protocol supplied by the manufacturer.

### 2.4. rfbE Gene Amplification of E. coli O157:H7

For the feasibility of the designed *rfbE* gene primers and the amplification production length of the primers, the conventional PCR reaction combined with 2% agarose gel electrophoresis was performed in sterile 0.2 mL eight-strip tubes. Briefly, the synthesized primers were diluted to 10 μM. The PCR reaction system was as follows: 2.0 μL of dNTP, 2.5 μL of 10 × PCR buffer (Mg^2+^), 0.2 μL of TaqDNA polymerase, 1.0 μL of DNA template, 1.0 μL of the upstream (D-F) and downstream (D-R) primers. The total PCR reaction volume was adjusted to 25 μL with aseptic ultrapure water. The temperature profile of the PCR reaction had an initial denaturation step of 95 °C for 5 min followed by 30 cycles (30 s at 94 °C, 30 s at 60 °C, 30 s at 72 °C) and extended at 72 °C for 6 min in a PCR thermal cycler (BIORAD, Shanghai, China). Then, 2% agarose gel electrophoresis was performed as follows. Firstly, 2% (Wt/Vol) agarose gel was formulated with TAE buffer solution (Dongsheng Biotech, Co., Ltd., Guangdong, China). After heating for 4 min with 2% gel prepared in a TAE buffer solution, the Golden View dye was added at a ratio of 1:20,000 when it was cooled to 60–70 °C. The solution was poured into a glue mold (Liuyi, Co., Ltd., Beijing, China) and cooled down to room temperature. Secondly, The PCR product sample was uniformly mixed with the 5x loading buffer in a ratio of 1:4. Finally, the samples were added into the gel hole, and gel electrophoresis was performed at 90 V for 45 min and visualized under UV light (BIORAD, Shanghai, China).

### 2.5. Theoretical Evaluation of the Target Gene Probe Performance

The real-time fluorescent PCR reaction was performed in 25 μL of a reaction mixture comprised of the following: 2.0 μL of dNTP, 2.5 μL of 10 x PCR buffer (Mg^2+^), 0.2 μL of TaqDNA polymerase, 1 μL of template DNA, 0.5 μL of TaqMan probes, 1 μL of the upstream (D-F) and downstream (D-R) primers and 16.8 μL of aseptic ultrapure water. Briefly, the real-time fluorescent PCR reaction was followed by monitoring the fluorescence probe change in real-time and carried out in three steps: initial denaturation at 95 °C for 3 min followed by 40 cycles (20 s at 95 °C, 20 s at 60 °C) and the reaction was extended at 72 °C for 30 s (the ramp speeds of the PCR steps was 0.5 °C/s) in a BIO-RAD CFX series real-time fluorescent PCR instrument (BIORAD, Shanghai, China).

### 2.6. Optimization of the Real-Time Fluorescent PCR Reaction System

The optimization of conditions was carried out to improve the analytical sensitivity, specificity, as well as to achieve a lower detection limit of the real-time fluorescent PCR reaction. For this purpose, the concentrations of primers and probes, as well as their annealing temperature were optimized as follows.

#### 2.6.1. Volume Improvement of Primer Addition

To find the best primer concentration for the real-time fluorescent PCR reaction system, the volume of the TaqMan probe B was determined as 0.5 μL, and A was set as 0.4, 0.6, 0.8, 1.0, 1.2, 1.4, 1.6 in turn. Next, it was well blended with other components (except the primer) shown in Table 2. At the same time, nuclease-free water instead of DNA extract was used as the no template or negative control. The temperature profile of the real-time fluorescent PCR reaction had an initial denaturation step at 95 °C for 3 min followed by 40 cycles (20 s at 95 °C, 20 s at 60 °C, the ramp speeds of the PCR steps was 0.5 °C/s) and extended at 72 °C for 30 s.

#### 2.6.2. Determination of the TaqMan Probe Dosage

The optimum probe volume of B for the TaqMan probe was evaluated at 0.3, 0.5, 0.7, 0.9, 1.1, and 1.3 μL. Subsequently, the next procedure was consistent with the reaction procedure in Section 2.6.1.

#### 2.6.3. Annealing Temperature Optimization of the Reaction System

The reaction system was studied with various annealing temperatures (64.8 °C, 64.4 °C, 63.5 °C, 61.9 °C, 60.0 °C, 58.4 °C, 57.3 °C, and 56.8 °C). The DNA template was replaced with aseptic ultrapure water for the negative control. The later processing profile was consistent with temperature profile in Section 2.6.1.

### 2.7. Assay Specificity of the Target Fragment Primer in Real-Time PCR

The specificity of the designed primers for the target fragment was tested. The primers were used to amplify other bacterial DNA such as *E. coli* O157:H7, *S. aureus*, *Salmonellas* well as *Listeria monocytogenes*. The specificity of the primers for the target fragment was confirmed by observing the amplification curves. The temperature profile of the real-time fluorescent PCR reaction was set for denaturation at 95 °C for 3 min followed by 40 cycles (20 s at 95 °C, 20 s at 60 °C. the ramp speeds of the PCR steps was 0.5 °C/s) and extended at 72 °C for 30 s.

### 2.8. Preparation and Optimum Concentration of IAC

The IAC was constructed using PCR as previously reported [21], and the influence of the amount of IAC was evaluated. *Listeria monocytogenes* was cultured overnight in a shaking bed (200 rpm) at 37 °C until the density of bacteria increased to 10^8^ CFU/mL. The DNA template was extracted from a 1-mL solution of *Listeria monocytogenes* via the boiling method and amplified by PCR. The IAC preparation involved two PCR amplifications [25]. The first step of PCR amplification employed a total reaction volume of 25 μL: 1 μL of DNA template,2 μL dNTP, 2.5 μL 10x PCR buffer (Mg^2+^), 1 μL of the upstream (IAC-F, 10 μmol/L) heterozygous primers, 1 μL of downstream (IAC-R, 10μmol/L) heterozygous primers. The PCR reaction conditions used were a denaturation step at 94 °C for 5 min, followed by 30 cycles (94 °C for 30 s, 60 °C for 30 s, 72 °C for 30 s) and extended at 72 °C for 6 min. The amplicon obtained from the first PCR was recovered following gel electrophoresis and purified by a DNA gel extraction kit according to the kit instructions. The second step of PCR amplification was to dilute the purified product from the first step at a ratio of 1:1000 with nuclease-free water. The diluted product (3 μL) was further amplified by PCR reaction. The procedure of the second PCR reaction, which confirmed that the first round of PCR product (hybrid DNA) contained the target primers [26], was the same as that of the first step PCR.

To select the smallest amount of positive fluorescence signals, the artificially created DNA was used as an internal amplification control (IAC) in every reaction mixture. The different volumes of IAC (0.3 μL, 0.6 μL, 0.9μL, 1.0 μL, 1.2 μL, 1.5 μL, 1.8 μL, and 2.1 μL) were added to the reaction system which did not contain the *E. coli* O157:H7 DNA. The IAC was detected by the real-time PCR assay using the probe.

### 2.9. Analytical Sensitivity of the Real-Time Fluorescent PCR Detection of E. coli O157:H7

A 10-fold bacterial suspension dilution series ranging from 10^8^ CFU/mL down to 10^1^ CFU/mL for *E. coli* O157:H7 and including a negative control, was prepared in duplicate. DNA of *E. coli* O157:H7 (1 mL) was respectively extracted by the boiling water method and by the commercial kit method. For each method, the changes of the fluorescence signal were measured in real-time during amplification using a BIO-RAD CFX fluorescent detector. To analyze the PCR data, the threshold (the minimal fluorescence which the signal of sample was detected by the fluorescence detector) was calculated. Subsequently, the Ct-value for each PCR sample was determined by the threshold from each PCR method. In addition, the analytical sensitivity of this technique was also compared with that of conventional PCR.

### 2.10. Detection of Simulated Drinking Water Samples

Simulated drinking water samples were prepared to test the applicability of IAC. The bacterial suspension of overnight culture, which reached 4.0 × 10^8^ CFU/mL, was carried out in a 10-fold dilution series with saline. After centrifugation for 5 min at 8000× *g* at room temperature, the supernatant was removed, and then the sediment at the bottom was retained and resuspended with the drinking mineral water. The final concentration of *E. coli* O157:H7 in drinking mineral water was 10^8^ CFU/mL, 10^7^ CFU/mL, 10^6^ CFU/mL, 10^5^ CFU/mL, 10^4^ CFU/mL, 10^3^ CFU/mL, 10^2^ CFU/mL, 10^1^ CFU/mL, respectively. DNA was extracted by using the boiling water method. The IAC probes were added to the fluorescent quantitative PCR system to test the applicability of IAC in simulated drinking water samples.

## 3. Results

### 3.1. Amplification of E. coli O157:H7 rfbE Gene

**Figure 1:**
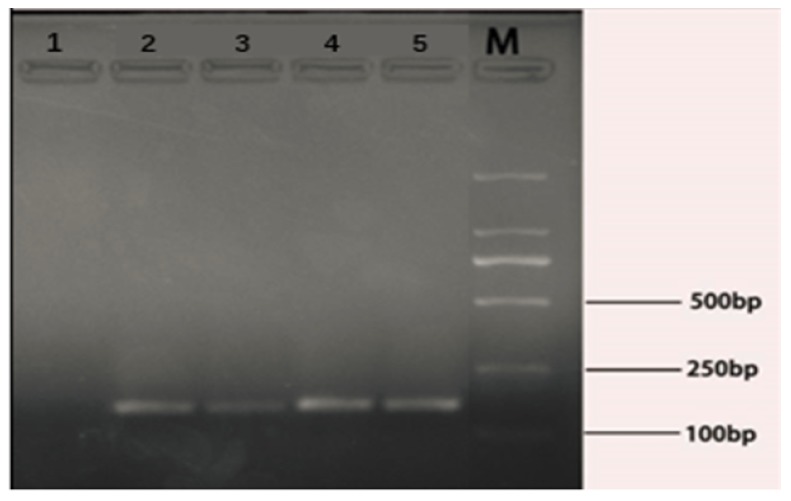
Amplification results of the *Escherichia coli* O157:H7 *rfbE* gene. Lane 1: ultrapure sterile water replaced the DNA template as a negative control. The lane 2, 3, 4 and 5 were four parallel samples of target gene amplification. Lane M: DL2000 DNA ladder.

The PCR amplification products were submitted for electrophoresis on 2% agarose gel to verify the validity of the target gene primers. The agarose gel electrophoresis showed the presence of target products (152 bp) with the PCR product size between 100 bp and 250 bp (Figure 1). The result was in agreement with the length (152 bp) of our predesigned primer product.

### 3.2. Validation of the Effectiveness of the Target Gene Probe

**Figure 2:**
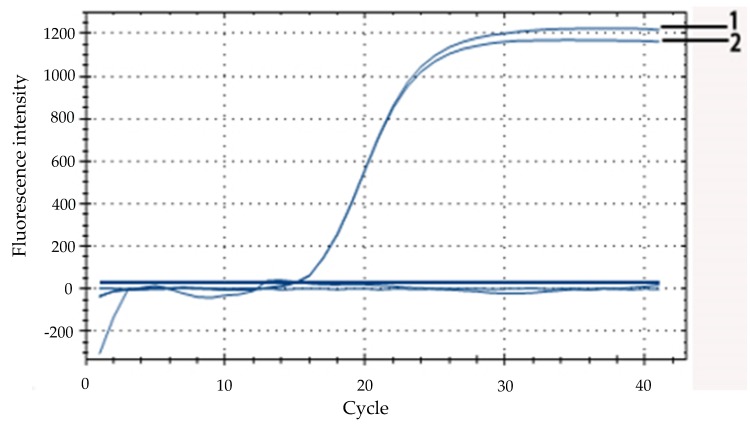
Validation of the effectiveness of the target gene probe. The curves 1 and 2 represent two parallel experiments to verify the validity of the TaqMan probes.

After 40 cycles in fluorescence quantitative PCR, two ideal amplification curves, where the fluorescence intensity was above 1000, were obtained. Moreover, the difference in C_t_ value was less than one cycle, which showed that the amplification efficiency of the probe was relatively high (Figure 2). The results demonstrated that the probe designed in this experiment was effective and feasible, and the primers and probes for the target species were highly specific.

### 3.3. Optimization of Real-Time Fluorescence PCR Reaction System

#### 3.3.1. Improvement of Primer Volume for *rfbE* Gene

**Figure 3:**
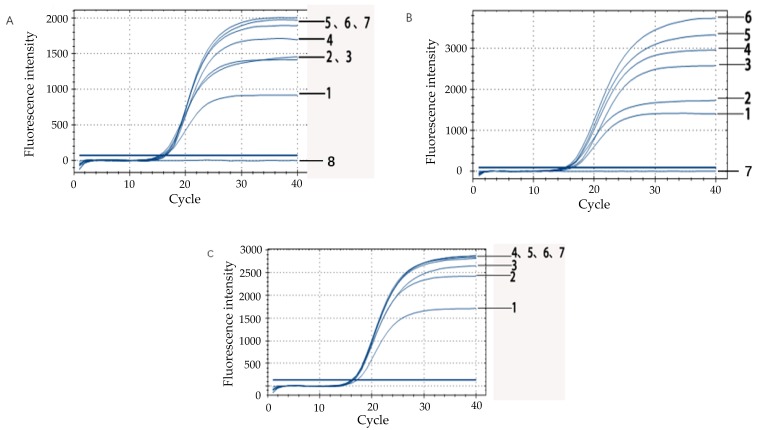
Optimization of real-time fluorescence PCR reaction system. (**A**) Volume improvement of primer based on the *rfbE* gene. Line 1: 0.4 μL; Line 2: 0.6 μL; Line 3: 0.8 μL; Line 4: 1.0 μL; Line 5: 1.2 μL; Line 6: 1.4 μL; Line 7: 1.6 μL. Line 8: negative control (PCR reaction system without primer) (**B**) Optimization of probe concentration of target gene in the system. Line 1: 0.3 μL; Line 2: 0.5 μL; Line 3: 0.7 μL; Line 4: 0.9 μL; Line 5: 1.1 μL; Line 6: 1.3 μL; Line 7: negative control (PCR reaction system without probe) (**C**) Annealing temperature optimization of the real-time fluorescence PCR reaction system. Line 1: 64.4 °C; Line 2: 57.3 °C; Line 3: 64.8 °C; Line 4: 61.9 °C; Line 5: 60 °C; Line 6: 58.4 °C; and Line 7: 63.5 °C.

Below 1.0 μL of the *rfbE* primers, the fluorescence intensity gradually decreased and was proportional to the decrease in primer volume. Above 1.0 μL of the *rfbE* primers, the fluorescence intensity signal was consistently maintained between 1500 and 2000 in the final plateau (Figure 3A). After comparing the fluorescence peaks and Ct values, 1.0 μL of the primer was considered as the most suitable for the real-time fluorescent PCR reaction system.

#### 3.3.2. Determination of the Amount of TaqMan Probe

As can be seen from the Figure 3B, the fluorescence signal of the amplification reaction appeared in each probe concentration, and there was no fluorescent signal in the negative control group (Figure 3B). The difference of Ct-values for each probe concentration was minimal. Taking into consideration the economic efficiency of the double-label probes and the maximum fluorescence intensity during the plateau, the optimum conditions for the probe was 10 μM, namely, the probe volume (0.5 μL) for the target gene was added to the final reaction volume (25 μL).

#### 3.3.3. Annealing Temperature Optimization of the Reaction System

By comparing the annealing times at different temperatures, we found that the difference in Ct values between 63.5 °C and 58.4 °C was less than one cycle. While the annealing temperature was too low, the specificity of the reaction was unfavorable. However, a high temperature resulted in the amplification inhibition of the target fragment when the annealing temperature of the primer and probe was taken into consideration. Finally, 60 °C was used as the most suitable annealing temperature when the Ct value was equal to 16.48.

### 3.4. Specific Detection of E. coli O157:H7 by Real-Time Fluorescent PCR

**Figure 4:**
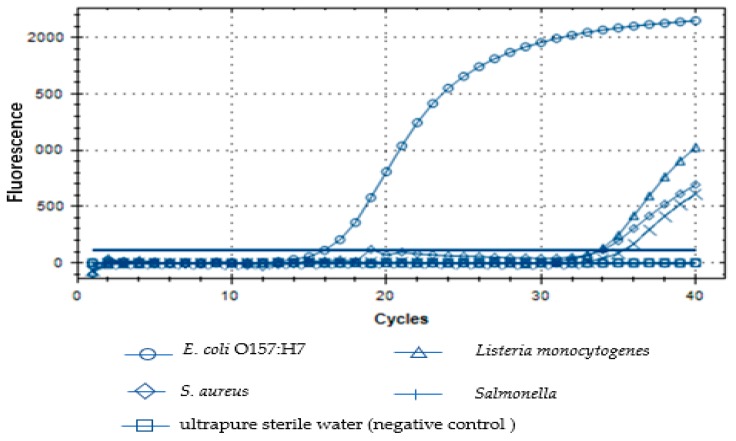
Specificity of the designed primers for the *rfbE* gene for *E. coli* O157:H7.

As shown in Figure 4, only DNA from *E. coli* O157:H7 produced a positive fluorescent signal that showed a typical fluorescent amplification curve when the C_t_ values was 16. Simultaneously, there was no amplification curve from the other bacteria tested, which indicated that the primers of the target gene were highly specific. It was concluded that real-time fluorescent PCR can specifically detect *E. coli* O157:H7.

### 3.5. The Preparation and Optimum IAC Volume for PCR Amplification

**Figure 5:**
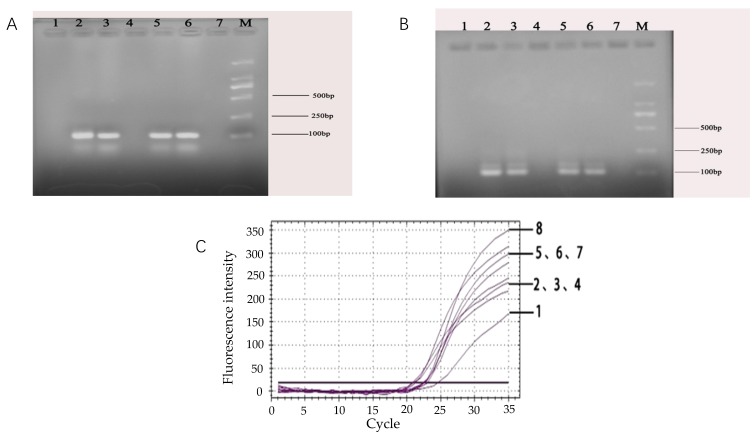
Products of the first step of PCR amplification. (**A**) Lane 2, 3, 5 and 6 showed the products prepared in the first PCR step. Lane M: DL 2000 ladder. (**B**) The second step of PCR amplification products. Lanes 2, 3, 5 and 6 showed the products prepared in the second PCR step. Lane M: DL 2000 ladder. (**C**) Determination of the optimum addition of IAC. Line 1: 0.3 μL; Line 2: 2.1 μL; Line 3: 1.8 μL; Line 4: 1.5 μL; Line 5: 1.2 μL; Line 6: 1.0 μL; Line 7: 0.9 μL; and Line 8: 0.6 μL.

The concentration of IACs appeared to be critical. Too much IAC DNA template would out-compete the target DNA template, thus giving a false negative result. However, the use of an optimal IAC concentration increased the reliability of the PCR assays and appeared to be useful for sample analysis. In this work, the IAC product was prepared by the first PCR. The results of the gel electrophoresis band were consistent with the theoretically designed 105 bp products, which demonstrated that the designed IAC was effective (Figure 5A). The PCR products from the second step were also in accordance with the experimental design requirements. Results showed that the first PCR products already contained the hybrid DNA of the target primer (Figure 5B).

It was shown in Figure 5C that high volumes of the IAC inhibited the amplification signal of the target DNA. Whereas, the lowest IAC volume was not sufficient to produce the desired IAC amplification signal. Therefore, the results showed that the optimum loading of IAC was 0.6 μL in the reaction system.

### 3.6. Sensitivity Test of Real-Time Fluorescent PCR for Detection of E. coli O157:H7

**Figure 6:**
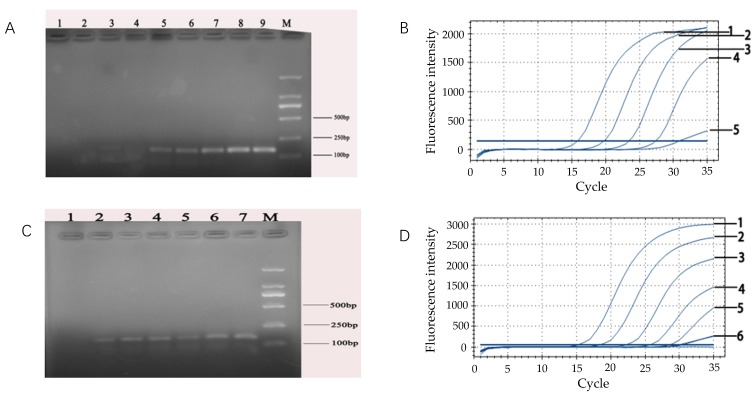
(**A**) Sensitivity of ordinary PCR was based on the different concentrations of *E. coli* O157:H7 DNA extracted by the boiling water method.Lane M: DL 2000 DNA ladder; Lane 9: 10^8^ CFU/mL; Lane 8: 10^7^ CFU/mL, Lane 7: 10^6^ CFU/mL, Lane 6: 10^5^ CFU/mL, and Lane 5: 10^4^ CFU/mL. (**B**) Sensitivity of the real-time fluorescent PCR based on different concentrations of *E. coli* O157:H7 DNA extracted by the boiling water method. Line 1: 10^8^ CFU/mL; Line 2: 10^7^ CFU/mL; Line 3: 10^6^ CFU/mL; Line 4: 10^5^ CFU/mL; and Line 5: 10^4^ CFU/mL. (**C**) Sensitivity of ordinary PCR based on different concentrations of *E. coli* O157:H7 DNA extracted by the commercial kit method. Lane M: DL 2000 DNA ladder; Lane 7:10^8^ CFU/mL; Lane 6: 10^7^ CFU/mL; Lane 5: 10^6^ CFU/mL; Lane 4: 10^5^ CFU/mL; Lane 3: 10^4^ CFU/mL; and Lane 2: 10^3^ CFU/mL. (**D**) Sensitivity of real-time fluorescent PCR based on different concentrations of *E. coli* O157:H7 DNA extracted by the commercial kit method. Line 1: 10^8^ CFU/mL; Line 2: 10^7^ CFU/mL; Line 3: 10^6^ CFU/mL; Line 4: 10^5^ CFU/mL; Line 5: 10^4^ CFU/mL; and Line 6: 10^3^ CFU/mL.

Figure 6 showed that the detection limit for *E. coli* O157:H7 by ordinary PCR was consistent with real-time fluorescence PCR. However, due to the degradation of DNA during thermal extraction, the purity of the amplified DNA extracted by the commercial kit was only 10-fold higher than the boiling water method (Figure 6A,C). The sensitivity of the system had been improved so that both the normal PCR and real-time PCR could detect 10^3^ CFU/mL *E. coli* O157:H7 (Figure 6). On the other hand, considering the results of efficiency, range of use, ease of use, and chemical toxicity, DNA extraction by the boiling water method was used in the subsequent experimental trials in simulated drinking water samples.

### 3.7. Sensitivity Detection of E.coli O157:H7 Spiked Drinking Water Samples

**Figure 7:**
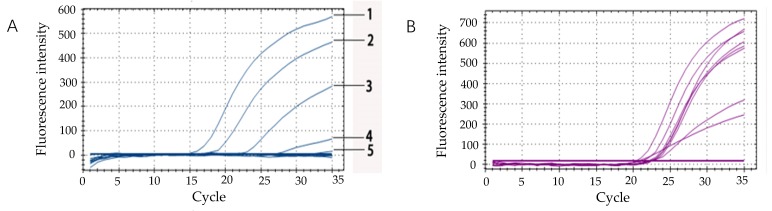
(**A**) Sensitivity test for detection of *Escherichia coli* O157:H7 in drinking Water by Real-time fluorescent PCR. Line 1: 10^8^ CFU/mL; Line 2: 10^7^ CFU/mL; Line 3: 10^6^ CFU/mL; Line 4: 10^5^ CFU/mL; and Line 5: 10^4^ CFU/mL. (**B**) Sensitivity test for detection of *Escherichia coli* O157:H7 in drinking Water when combining IAC with real-time fluorescent PCR.

In this experiment, water spiked with *E. coli* O157:H7 was used as a simulated sample. The sensitivity was reduced because some components in the water may affect the results of normal PCR. A fluorescence quantitative PCR method including competitive IAC was established to detect simulated water samples when DNA template for *E. coli* O157:H7 was also extracted by the boiling water method. Figure 7 shows that the positive IAC signals appeared in every sample when the sample concentrations were higher than 10^4^ CFU/mL. It demonstrated that the lowest detection limit of *E. coli* O157:H7 in water samples was 10^4^ CFU/mL. Moreover, the best IAC positive signals were presented when the concentration of the bacteria solution was between 10^5^ CFU/mL and 10^8^ CFU/mL. The appearance of an IAC signal implied that the false negative results were excluded. Furthermore, the sensitivity was consistent with that in the pure culture experiment in that it showed that fluorescent quantitative PCR, including competitive IAC, was reliable for excluding negatives in when detecting *E. coli* O157:H7 in food samples.

## 4. Discussion

*E. coli* O157:H7 is one of the three major foodborne pathogenic bacteria in the world. When the intake reaches a certain amount, public health will be threatened. Once an outbreak reaches epidemic proportions, it will cause an inestimable economic burden [27]. Until now, with the continuous development of PCR technology [28], fluorescent quantitative PCR has been widely used for the detection of foodborne pathogens. The most precise, accurate and sensitive real-time PCR methods were all TaqMan-based methods rather than molecular beacon-based methods. Nevertheless, the precision, accuracy, and sensitivity of a PCR-based method are not defined by the choice of detection probe, but mainly by the PCR performance itself (which is influenced by the primer sequence, primer specificity, annealing temperature, etc.). The fluorescent quantitative PCR can monitor the amplification process through fluorescence. However, despite the improvement of PCR technology, false-negative results in PCR detection remain as an unresolved issue, which may reduce the detection accuracy [29]. To address this challenge, an internal amplification control (IAC), which is a non-target DNA fragment introduced into the PCR detection system and is co-amplified with the target sequence so as to exclude false negative results produced by the PCR inhibitors in the samples.

Cankar et al. affirmed that the presence of large amounts of background DNA could also have effect on the target DNA amplification [30]. It is generally assumed that changes in PCR efficiency may occur due to extraneous substances in the isolated DNA, such as enhancers or inhibitors of the PCR reaction, originating either from the sample matrix or from the DNA extraction solution [30]. Some methods showed an important reduction of the analytical sensitivity when samples were tested in comparison to purified DNA, suggesting that the DNA purification step was crucial for the PCR yield. Since most procedures lacked internal amplification controls, discrimination between true and false negative results could often not be assessed. Indeed, PCR cannot illuminate diagnostic results before it includes an internal amplification control. So it is necessary to use IAC with real-time PCR detection to identify false negative results and to control for the presence of amplification inhibitors via different fluorescent signals emitted by their respective specific probes, especially for certified routine diagnostic laboratories. Further, Jebbink et al. [31] found no difference in accuracy and reliability between real-time PCR assays using TaqMan probes and molecular beacons for quantitative analysis of Epstein-Barr virus and Cytomegalovirus. Thus, using molecular beacons or TaqMan probes for real-time PCR detection will not affect the sensitivity, precision or accuracy of the method.

In previous reports, a non-competitive IAC with two pairs of primers being complementary to both the target DNA and non-target DNA was used to indicate PCR inhibition. However, the inclusion of additional primer sets in the reaction invariably results in a higher probability of mis-priming and primer dimerization [32]. It is difficult to avoid the interference among primer sets and reaction conditions for all primer sets must be optimized. The purpose of this paper is to establish a real-time fluorescence PCR detection system for detection of *E. coli* O157:H7 by adding a competitive internal amplification control (IAC) which could indicate the false-negative results without the decrease of detection sensitivity [33]. But, due to the co-amplification of the IAC, a reduction in target sensitivity was observed. This is not surprising, since such a reduction is inherent to the simultaneous amplification of different targets in one reaction. However, introducing co-amplification of the IAC has only a minor influence on target sensitivity.

Furthermore, in this study, the DNA of *E. coli* O157:H7 in pure culture medium was extracted by both the water-boiling method and commercial kit. The results showed that the detection limit of DNA extracted by the commercial kit (10^3^ CFU/mL) was higher (10-fold) than the sensitivity of DNA extracted by boiling method (10^4^ CFU/mL). With regard to the boiling method, it has the advantages of simple operation, low cost and strong economic applicability although water-boiling is time-consuming. On the other hand, the DNA template with higher purity can be extracted by the kit extraction method which can be used for the detection of complex samples. Meanwhile, the results showed that the fluorescence quantitative PCR method established in this paper is consistent with the traditional PCR method. It demonstrated that the real-time monitoring of the process does not require post-amplification treatment of the samples, such as gel electrophoresis, which reduces the time of analysis and risk of cross-contamination [16]. The detection limit of DNA extraction is in line with Teegan et al. [34] who indicated that the PVPP spun columns and the UltraClean kit had the best detection limit, detecting 20 pg of *E. coli* DNA (about 2 × 10^3^ cells) per 100 mg of manure.

The method of using the Ct values of IAC products from all samples tested in every PCR run was carried out to validate qPCR results, the detect outlier values of IAC-PCR would indicate poor DNA yield or inhibition, leading to a false negative result. In this work, the sensitivity of the *E. coli* O157:H7 detection limit was not changed after the introduction of IAC. At the same time, an IAC positive signal appeared in each sample, indicating that the false positive result was absent during the PCR reaction. Interestingly, our finding was quite similar to the study of Wang et al. [35], showing a detection limit of 1.2 × 10^3^ CFU/mL in pure culture as well as infant formula. However, this newly developed qRT-PCR assay reduced the cycles from 30 to 20 compared with Seo and Brackett [36], preventing the self-degradation and fluorescent signal release after 30 cycles. The Ct-values of the IAC were stable between 20 and 25 cycles in simulated drinking water samples. Therefore, our results indicated that the qRT-PCR assay could detect *E. coli* O157:H7 from samples and avoid false negatives by using an internal amplification control.

In conclusion, an IAC control, of competitive traits, was constructed for an *E. coli* O157:H7 PCR assay to monitor the amplification. The optimized real-time PCR detection with IAC for *E. coli* O157:H7 may provide an improved, sensitive, precise, and accurate method. It is applicable for rapid analysis and routine diagnosis, preventing false negative reactions and providing a tool for the accurate quantification of *E. coli* O157:H7.

## 5. Conclusions

In this study, the real-time PCR detection system using a competitive IAC with a target gene presented a highly specific (only positive for *E. coli* O157:H7), sensitive (10^3^~10^4^ CFU/mL of purified genomic DNA), and faster (compared to traditional cultural method) method for the detection of *E. coli* O157:H7 in water samples. It ensures the low homology between the amplified internal target and the target gene so that the two sequences would not affect the detection sensitivity through the combination of complementary chains, and the NCBI search was used to ensure the extremely low homology between the IAC probe and the target species DNA. These sequences were constructed into a competitive amplification internal standard to avoid the interference with primers in the PCR reaction system. Different fluorescent group probes were used to amplify the internal standard and target genes, and various different fluorescent signals were emitted during the reaction so as to achieve the purpose of indicating false negatives. This study indicated that the real-time fluorescent PCR-IAC method established in the experiment was more applicable and reproducible for eliminating false negatives in food samples.

The optimized methods for the detection of *E. coli* O157:H7 with introducing the IAC described in this study are useful tools for the analysis of target DNA. However, in this paper only DNA extracted from pure cultures was tested. The detection of *E. coli* O157:H7 in substrates like fruit, meat, or poultry might introduce a reduction in sensitivity, precision, or accuracy. Therefore, the general applicability of IAC-PCR technology in different samples needs further confirmation.

## Figures and Tables

**Table 1:** The primers and TaqMan probes used for PCR.

Target Gene	Name	Sequence (5′-3′)	Product Length (bp)
*rfbE*	D1-F	AACTAGGACCGCAGAGGAAAGAG	158
	D2-R	CACGCCAACCAAGATCCTCA	
	D-P	TGCAGATAAACTCATCGAAACAAGGCC	
IAC	IAC-F	AACTAGGACCGCAGAGGAAAGAGCATGGCACCAGCATCT	105
	IAC-R	CACGCCAACCAAGATCCTCAATCCGCGTGTTTCTTTTCGA	
	IAC-P	Cy5-CGCCTGCAAGTCCTAAGACGCCA- BHQ2	

F, R denote forward and reverse primers, respectively, P denotes the specific TaqMan probes corresponding to *rfbE* gene and IAC. Cy5 and BHQ2 are the 5’ end labeled report group and 3’ end quenching group of the IAC TaqMan probe, separately.

**Table 2:** Real-time fluorescent PCR reaction system.

dNTP	2.0 μL
10 x PCR buffer (Mg^2+^)	2.5 μL
D-F	A μL
D-R	A μL
TaqDNA polymerase	0.2 μL
template DNA	1.0 μL
TaqMan probes	B μL
aseptic ultrapure water	Constant volume to 25 μL

A and B represent the volume of the primers and probes designed for the *E. coli* O157:H7 *rfbE* gene.
